# Establishment of risk model for elderly CAP at different age stages: a single-center retrospective observational study

**DOI:** 10.1038/s41598-023-39542-3

**Published:** 2023-08-01

**Authors:** Chunxin Lv, Teng Pan, Wen Shi, Weixiong Peng, Yue Gao, Abdul Muhith, Yang Mu, Jiayi Xu, Jinhai Deng, Wei Wei

**Affiliations:** 1 Oncology Department, Shanghai Punan Hospital of Pudong New District, No 279, Linyi Road, Pudong, Shanghai, China; 2 Longgang District Maternity & Child Healthcare Hospital of Shenzhen City, Shenzhen, China; 3 Faculty of Life Sciences and Medicine, School of Cancer and Pharmaceutical Sciences, King’s College London, London, UK; 4 Department of Dermatology, Shanghai Punan Hospital of Pudong New District, No 279, Linyi Road, Shanghai, China; 5 Hunan Zixing Artificial Intelligence Technology Group Co., Ltd., Hunan Province, Changsha City, China; 6 Department of Oncology, Royal Marsden Hospital, London, UK; 7 Geriatric Department, Minhang Hospital, Fudan University, No 170, Xinsong Road, Shanghai, China; 8 Richard Dimbleby Department of Cancer Research, Comprehensive Cancer Centre, Kings College London, London, SE1 1UL UK; 9 Clinical Research Center (CRC), Medical Pathology Center (MPC), Cancer Early Detection and Treatment Center (CEDTC), Translational Medicine Research Center (TMRC), Chongqing University Three Gorges Hospital, Chongqing University, Wanzhou, Chongqing, China

**Keywords:** Health care, Risk factors

## Abstract

Community-acquired pneumonia (CAP) is one of the main reasons of mortality and morbidity in elderly population, causing substantial clinical and economic impacts. However, clinically available score systems have been shown to demonstrate poor prediction of mortality for patients aged over 65. Especially, no existing clinical model can predict morbidity and mortality for CAP patients among different age stages. Here, we aimed to understand the impact of age variable on the establishment of assessment model and explored prognostic factors and new biomarkers in predicting mortality. We retrospectively analyzed elderly patients with CAP in Minhang Hospital, Fudan University. We used univariate and multiple logistic regression analyses to study the prognostic factors of mortality in each age-based subgroup. The prediction accuracy of the prognostic factors was determined by the Receiver Operating Characteristic curves and the area under the curves. Combination models were established using several logistic regressions to save the predicted probabilities. Four factors with independently prognostic significance were shared among all the groups, namely Albumin, BUN, NLR and Pulse, using univariate analysis and multiple logistic regression analysis. Then we built a model with these 4 variables (as ABNP model) to predict the in-hospital mortality in all three groups. The AUC value of the ABNP model were 0.888 (95% CI 0.854–0.917, *p* < 0.000), 0.912 (95% CI 0.880–0.938, *p* < 0.000) and 0.872 (95% CI 0.833–0.905, *p* < 0.000) in group 1, 2 and 3, respectively. We established a predictive model for mortality based on an age variable -specific study of elderly patients with CAP, with higher AUC value than PSI, CURB-65 and qSOFA in predicting mortality in different age groups (66–75/ 76–85/ over 85 years).

## Introduction

Community-acquired pneumonia (CAP) is one of the main reasons of mortality and morbidity in elderly population, causing substantial clinical and economic impacts^1,2^. The elderly are inclined to pneumonia-associated death because of poor physical state, including weakened immune system, comorbidities, poor functional status, and dysphagia^3–6^, which makes elderly CAP even harder to cure. Thus, more attention should be paid on the introduction of accurate systems to predict the prognoses of elderly CAP patients as early as possible, and it is beneficial for early classification and further decrease the in-hospital mortality.

Clinically, there are several assessment tools widely used for predicting the hospital mortality of patients with CAP, including Confusion, Urea, Respiratory Rate, Blood Pressure, and Age ≥ 65 (CURB-65), Pneumonia Severity Index (PSI) and quick Sequential Organ Function Assessment (qSOFA)^7–9^. However, these score systems have been shown to demonstrate poor prediction of mortality for patients aged over 65. For example, Song et al*.* showed that PSI value (AUC = 0.576) was not a reliable prognostic predictor in elderly patients (aged ≥ 65 years) with CAP^10^. Similarly, another study reported the AUC values of CURB-65 and qSOFA were only 0.65 and 0.64, respectively, in predicting the mortality of elderly patients with a median age of 81 years (IQR 67–90)^11^. Consistently, Baek et al*.* also revealed the predictive performances of the CURB-65 and PSI were not ideal in high-aged patients (aged 80 or over with pneumonia, with AUC just being 0.61 and 0.52, respectively^12^. In contrast, it is reported that the AUC values of PSI and CURB-65 models in predicting mortality in young population (aged 18–64 years) were 0.87 and 0.73, respectively, significantly higher than that in elderly population (aged 65 or over)^13^. Therefore, the establishment of a new efficient tool to predict prognosis of elderly CAP patients is in unmet clinical need. Furthermore, in CAP patients over 65, there were still variations in the prediction of morbidity and mortality by just one available model because CAP patients of different age groups demonstrated different clinical outcomes^14^. However, few studies have focused on the finding of prognostic factors in predicting mortality of elderly patients with CAP accurately in different age subgroups.

In this study, we aimed to understand the impact of age on assessment model establishment and conducted an age variable-specific study of elderly patients with CAP to explore prognostic factors and new biomarkers in predicting mortality. We divided enrolled patients into three groups according to age variable: aged 66–75 years group (including 415 patients), aged 76–85 years group (394 patients enrolled), and aged over 85 years group (containing 365 patients), respectively. As a result, we found four variables with significance among all the groups of different age stage (66–75/ 76–85/ over 85 years), including Albumin, BUN, NLR and Pulse. Notably, the AUCs in predicting mortality in these three groups by the four variables were higher than PSI, CURB-65 and qSOFA.

## Materials and methods

### Research Objects

The study was approved by the Ethics Committee of the Minhang Hospital, Fudan University in Shanghai, China (Lot No: Medical Ethics Committee (2017) No. 42). We retrospectively enrolled patients aged over 65 years with CAP between January 1, 2018, to September 1, 2022. Informed consents were obtained from patients or their legal guardians who agreed to participate in the study. Signatures of study population were obtained, and all procedures are in accordance with the Declaration of Helsinki.

The inclusion criteria were: (1) Age > 65 years; and (2) Diagnosed with CAP based on Chinese clinical practice guideline for community-acquired pneumonia (CAP) in adults^15^. The exclusion criteria were: (1), Immunosuppression such as corticosteroids (> 14 days), immunosuppressed individual, eg, HIV-positive, receiving chemotherapy or radiotherapy within 90 days and transplant recipients; and (2) Patients with healthcare-associated pneumonia (HCAP).

### Data collection

The following clinical data within 24 h of admission to Minhang Hospital, Fudan University were collected anonymously from electronic medical records: demographics, smoking, dysphagia, comorbidities, primary symptoms, vital signs on admission and prognosis, as well as laboratory variables (hematological data, biochemical parameters, coagulation indicators, inflammatory markers, imaging examination, etc.).

The CURB-65 (confusion, urea > 7 mmol/L, respiratory rate ≥ 30/min, systolic blood pressure < 90 mmHg or diastolic blood pressure ≤ 60 mmHg, age ≥ 65 years), qSOFA (systolic blood pressure ≤ 100 mmHg, respiratory rate ≥ 22/min, and altered cognitive state) and PSI (demographics, comorbidities, a physical examination, and laboratory and radiological findings) were measured and recorded^16–18^.

### Statistical analysis

MedCalc software (version 20.1.0) was used for statistical analysis. Data with normal distribution were described as mean ± standard deviation and compared using Student's *t*-test. Data with skewed distribution were expressed as median (Inter-Quartile Range) and compared using the Mann–Whitney *U* nonparametric test. The classification variables were presented as percentages and compared using the Chi-squared test or Fisher's exact test. Multivariate analysis using stepwise logistic regression analysis was used to evaluate all parameters with *P* value < 0.05 in univariate analysis. The prediction accuracy of the prognostic factors was determined by the Receiver Operating Characteristic (ROC) curves and the area under the curves (AUC). Combination models were established using several logistic regressions to save the predicted probabilities. ROC curve analysis was performed using the saved probabilities as a new indicator. *P* value < 0.05 was considered statistically significant.

### Institutional review board statement

The study was conducted in accordance with the Declaration of Helsinki, and approved by the Ethics Committee of the Minhang Hospital, Fudan University in Shanghai, and the Lot No: Medical Ethics Committee (2017) No. 42.

### Ethics and informed consent statement

This study was approved by the Ethics Committee of the Minhang Hospital, Fudan University in Shanghai, and the Lot No: Medical Ethics Committee (2017) No. 42. Informed consent was obtained from all subjects involved in the study. Written informed consent has been obtained from the patient(s) to publish this paper.

## Results

### Clinical characteristics of patients in different age groups

After selection based on age variable and other exclusion criteria, a total of 1174 CAP patients over 65 years old (ranging from 66 to 107 years, with an average of 79 years old) were enrolled in this study (Supplementary Fig. 1). The study cohort was divided into three groups classified by age variable: group 1 (aged 66–75 years group with 415 patients, group 2 (aged 76–85 years group with 394 patients, and group 3 (aged over 85 years group containing 365 patients.

**Figure 1:**
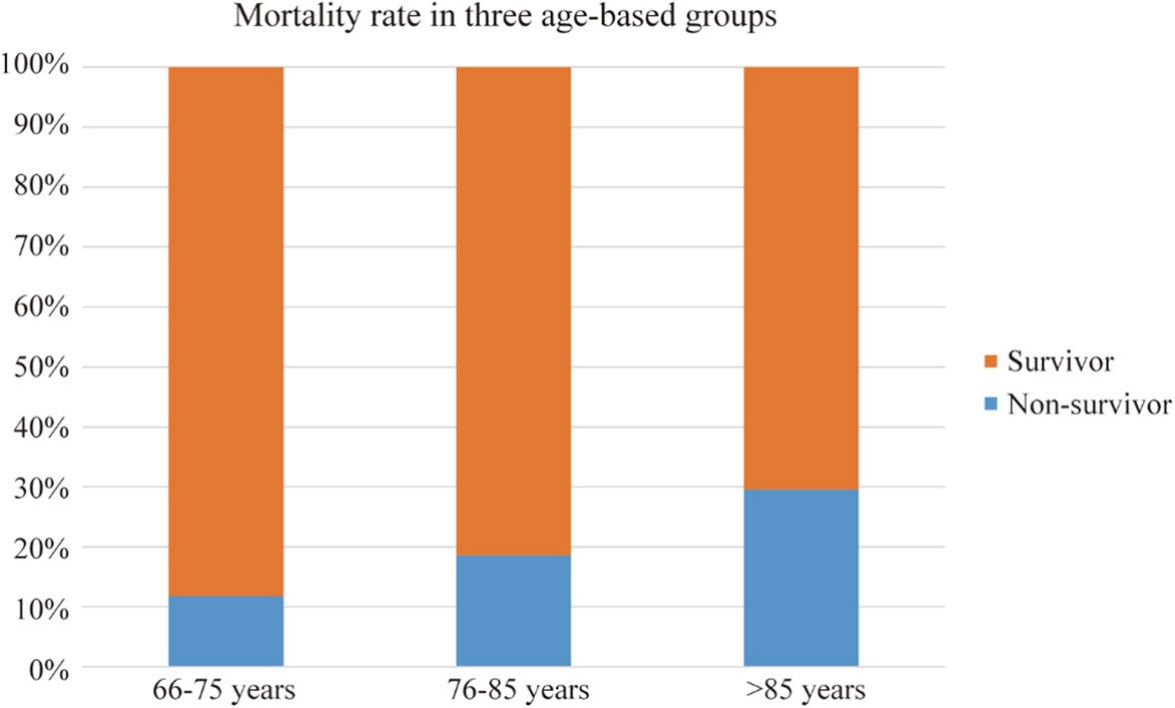
Mortality rate in three aged-based groups. The mortality rates were 11.81%, 18.53% and 29.59% in three age-based groups, respectively.

In these three age-based groups classified above, we divided each group into two populations based on clinical outcome, namely survivor group and non-survivor group. The mortality rate was 11.81% (49/415) in group 118.53% (73/394) in group 2, and 29.59% (108/365) in group 3, respectively (Fig. 1). Next, we analyzed clinically related factors and found out that different variables showed significantly different impacts between survivor group and non-survivor group across the groups classified by age intervals. Specifically, the variables in group 1 included gender, pulse, systolic pressure, leucocyte count, neutrophils count, lymphocyte count, neutrophil-to-lymphocyte ratio (NLR), c-reactive protein (CRP), procalcitonin(pct), albumin, urea nitrogen (BUN), D-dimer and cancer(*p* < 0.05). But in group 2, the statistically significant parameters, which were associated with the prognosis of CAP, contained age, pulse, systolic pressure, diastolic pressure, respiratory rate, leucocyte count, neutrophils count, lymphocyte count, NLR, CRP, pct, albumin, prealbumin, BUN, D-dimer and electrolyte disturbance. Regarding group 3, the variables included pulse, systolic pressure, respiratory rate, leucocyte count, lymphocyte count, NLR, CRP, pct, albumin, low-density lipoprotein, BUN, D-dimer and comorbidities (electrolyte disturbance, cancer, chronic kidney disease, congestive heart failure, coronary heart disease, hypertension) (*p* < 0.05) (Table 1). Collectively, our results demonstrated that factors showing prognostic values vary among different age groups of the elderly CAP patients.

**Table 1:** Basic characteristics of three age-based group patients.

Predictive factors	Group 1 (66–75 y) (n = 415)			Group 2 (76–85 y) (n = 394)			Group 3 (> 85 y) (n = 365)		
	Survivor cohorts (n = 366)	Non-survivor cohorts (n = 49)	*p* value	Survivor cohorts (n = 321)	Non-survivor cohorts (n = 73)	*p* value	Survivor cohorts (n = 257)	Non-survivor cohorts (n = 108)	*p* value
Gender (male/female)	227/139	39/10	0.016	184/137	49/24	0.094	119/138	56/52	0.333
Age (years)	70 (68–73)	71 (69–73)	0.054	79 (77–82)	81 (79–83)	0.005	89 (86–92)	89.5 (87–92.5)	0.056
Smoking	90/366	17/49	0.258	57/321	13/73	0.993	26/257	15/108	0.356
Pulse (/min)	87.88 ± 17.32	101.76 ± 21.64	0.002	89.15 ± 13.43	97.12 ± 19.73	0.008	85.90 ± 16.89	97.47 ± 21.96	< 0.000
Systolic pressure (mmHg)	122 (110–137)	118 (107–133)	0.042	130 (120–140)	117 (100–140)	0.001	128 (110–143)	120 (101–140)	0.037
Diastolic pressure (mmHg)	70 (68–80)	75 (60–82)	0.889	72 (70–80)	69 (58–78)	0.000	70 (59–79)	70 (60–80)	0.189
Respiratory rate (/min)	20 (19–20)	20 (18–22)	0.053	20 (19–20)	21 (18–21)	0.040	19 (18–20)	20 (19–22)	< 0.000
Dysphagia	42/366	11/49	0.066	54/321	12/73	0.946	47/257	20/108	0.965
Leucocyte count (*10^9)	8.14 ± 4.32	12.51 ± 8.64	0.006	9.23 ± 3.41	13.97 ± 6.28	< 0.000	8.79 ± 4.56	12.32 ± 5.38	< 0.000
Neutrophils count (*10^9)	5.95 ± 3.95	10.53 ± 7.43	0.001	7.22 ± 3.41	12.56 ± 6.23	< 0.000	9.88 ± 4.47	10.66 ± 5.28	0.395
Lymphocyte count (*10^9)	1.22 (0.84–1.67)	0.88 (0.37–1.28)	0.001	1.13 (0.82–1.52)	0.69 (0.44–0.92)	< 0.000	1.16 (0.83–1.60)	0.72 (0.51–1.20)	< 0.000
Neutrophil-to-Lymphocyte Ratio (NLR)	4.68 (2.81–8.24)	10.69 (4.48–17.13)	< 0.000	5.41 (2.94–10.24)	16.49 (11.77–25.40)	< 0.000	4.53 (2.61–7.86)	13.65 (7.94–24.41)	< 0.000
Platelet count (*10^9)	223.01 ± 95.00	187.64 ± 93.72	0.018	221.50 ± 92.00	200.00 ± 95.21	0.148	202.70 ± 80.23	197.81 ± 98.94	0.778
C-reactive protein CRP (ug/ml)	61.76 ± 62.81	100.14 ± 80.22	0.022	62.78 ± 61.71	137.80 ± 102.51	< 0.000	71.65 ± 62.96	109.56 ± 75.32	0.000
Procalcitonin pct (ng/ml)	0.12(0.06–0.45)	1.35 (0.45–3.63)	< 0.000	0.16 (0.08–0.69)	1.60 (0.36–7.63)	< 0.000	0.35 (0.10–1.55)	0.99 (0.46–2.65)	< 0.000
Albumin (g/L)	34.49 ± 5.19	28.13 ± 4.89	< 0.000	33.59 ± 5.38	27.78 ± 4.62	< 0.000	32.80 ± 5.02	28.65 ± 4.49	< 0.000
Prealbumin (mg/L)	130.09 ± 56.83	112.18 ± 49.16	0.235	128.53 ± 58.50	94.78 ± 45.52	0.003	110.88 ± 41.46	112.71 ± 59.61	0.852
Low-density lipoprotein (mmol/L)	2.20 ± 0.71	2.36 ± 1.32	0.520	2.33 ± 0.71	2.16 ± 0.91	0.348	2.43 ± 0.99	1.89 ± 0.78	0.002
Urea nitrogen BUN (mmo/l)	5.00 (3.80–7.10)	9.82 (7.32–17.96)	< 0.000	5.70 (4.20–8.52)	12.3 (9.68–23.33)	< 0.000	5.50 (3.50–8.02)	11.80 (7.08–17.56)	< 0.000
D-dimer (mg/L)	1.23 (0.74–2.27)	4.55 (2.32–9.57)	< 0.000	1.41 (0.78–2.46)	5.26 (2.56–8.20)	< 0.000	1.85 (1.10–3.15)	4.10 (1.89–8.45)	< 0.000
Electrolyte disturbance	128/366	11/49	0.201	60/321	24/73	0.038	22/257	35/108	< 0.000
Cancer	30/366	14/49	0.000	20/321	5/73	0.855	8/257	10/108	0.020
Chronic kidney disease	60/366	7/49	0.747	19/321	6/73	0.498	10/257	15/108	0.001
Congestive heart failure	74/366	9/49	0.803	46/321	7/73	0.343	26/257	23/108	0.014
Cerebrovascular disease	91/366	14/49	0.669	80/321	15/73	0.533	58/257	30/108	0.410
Coronary heart disease	63/366	6/49	0.451	69/321	15/73	0.886	28/257	28/108	0.002
Hypertension	189/366	15/49	0.087	156/321	34/73	0.853	52/257	55/108	< 0.000
Diabetes	89/366	7/49	0.202	80/321	18/73	0.971	40/257	24/108	0.206

### Multiple logistic regression analysis

**Figure 2:**
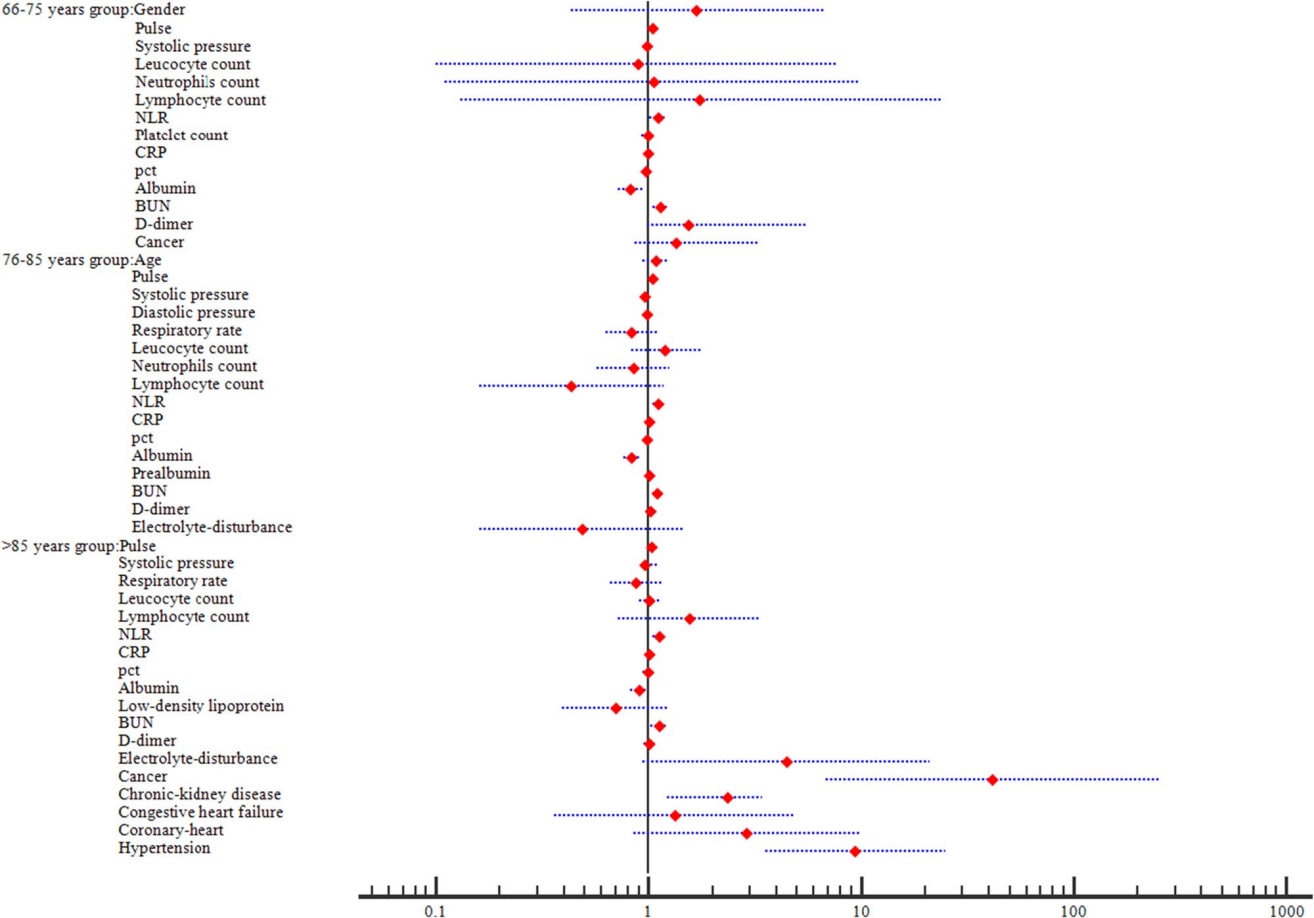
Forest plot of multivariate analysis in three age-based groups. The forest plot showed Pulse, NLR, BUN and Albumin were independent factors in group 1; five variables including Pulse, NLR, CRP, Albumin and BUN were demonstrated to be independently and statistically significant in group 2; And Pulse, NLR, Albumin, BUN, Cancer, Chronic-kidney and Hypertension were observed to independently influence the mortality in group 3.

To study the prognostic factors of mortality in each age-based group, we used those significantly altered variables from univariate analysis for multiple logistic regression model analysis. In group 1, the results showed that three factors were independent risk factors, including Pulse (*p* = 0.041, OR 1.036, 95% CI 1.001–1.072), NLR (*p* = 0.026, OR = 1.112, 95% CI 1.013–1.223) and BUN (*p* = 0.001, OR 1.135, 95% CI 1.051–1.226) were independent risk factors, whereas Albumin (*p* = 0.005, OR 0.825, 95% CI 0.723–0.942) was the only independent protective factor. In group 2, five variables were demonstrated to be independently and statistically significant predictors, including Pulse (*p* = 0.002, OR 1.039, 95% CI 1.014–1.065), NLR (*p* = 0.011, OR 1.115, 95% CI 1.050–1.212), CRP (*p* = 0.040, OR 1.005, 95% CI 1.001–1.010), Albumin (*p* < 0.000, OR 0.827, 95% CI 0.759–0.901), and BUN (*p* = 0.000, OR 1.098, 95% CI 1.043–1.156). In group 3, eight factors were observed to independently influence the mortality, involving Pulse (*p* = 0.030, OR 1.027, 95% CI 1.002–1.052), NLR (*p* = 0.001, OR 1.125, 95% CI 1.049–1.206), Albumin (*p* = 0.042, OR 0.905, 95% CI 0.823–0.997), BUN (*p* = 0.010, OR 1.117, 95% CI 1.026–1.215), Cancer (*p* = 0.000, OR 41.589, 95% CI 6.802–254.273), Chronic-kidney (*p* = 0.032, OR 2.34, 95% CI 1.23–3.41) and Hypertension (*p* < 0.000, OR 9.397, 95% CI 3.539–24.954) (Table 2, Fig. 2). Moreover, the R squared value were 0.320 in Group1, 0.353 in Group 2 and 0.409 in Group 3, respectively, confirming that the data were reliable. Thus, these data demonstrated four factors with independently prognostic value were shared among all the groups, namely Albumin, BUN, NLR and Pulse.

**Table 2:** Multivariate analysis for mortality in three groups.

Variables	Group 1		Group 2		Group 3	
	OR (95% CI)	*p*	OR (95% CI)	*p*	OR (95% CI)	*p*
Gender	1.686 (0.427–6.657)	0.455				
Age			1.077 (0.945–1.229)	0.266		
Smoking						
Pulse	1.036 (1.011–1.072)	0.041	1.039 (1.014 − 1.065)	0.002	1.027 (1.002–1.052)	0.030
Systolic pressure	0.980 (0.954–1.005)	0.117	0.963 (0.941 − 1.015)	0.492	0.963 (0.931–1.124)	0.056
Diastolic pressure			0.980 (0.950 − 1.011)	0.520		
Respiratory rate			0.836 (0.628–1.114)	0.221	0.872 (0.661–1.152)	0.335
Dysphagia						
Leucocyte count	0.885 (0.102–7.647)	0.911	1.204 (0.829–1.751)	0.330	1.013 (0.906–1.134)	0.814
Neutrophils count	1.059 (0.113–9.943)	0.960	0.847 (0.568–1.262)	0.414		
Lymphocyte count	1.744 (0.128–23.773)	0.676	0.436 (0.161–1.179)	0.102	1.550 (0.727–3.305)	0.256
NLR	1.112 (1.013–1.223)	0.026	1.115 (1.050–1.212)	0.011	1.125 (1.049–1.206)	0.001
Platelet count	0.998 (0.993–1.005)	0.736				
CRP	0.997 (0.989–1.005)	0.465	1.005 (1.001–1.010)	0.040	1.005 (0.998–1.012)	0.161
pct	0.976 (0.940–1.014)	0.206	0.989(0.962–1.017)	0.450	0.993 (0.949–1.039)	0.763
Albumin	0.825 (0.723–0.942)	0.005	0.827 (0.759–0.901)	< 0.000	0.905 (0.823–0.997)	0.042
Prealbumin			1.005 (0.997–1.014)	0.202		
Low-density lipoprotein					0.700 (0.392–1.247)	0.226
BUN	1.135 (1.051–1.226)	0.001	1.098 (1.043–1.156)	0.000	1.117 (1.026–1.215)	0.010
D-dimer	1.543 (0.985–5.587)	0.096	1.025 (0.973–1.080)	0.351	1.007 (0.947–1.071)	0.821
Electrolyte-disturbance			0.487 (0.161–1.472)	0.202	4.465 (0.943–21.141)	0.059
Cancer	1.342 (0.867–3.237)	0.998			41.589 (6.802–254.273)	0.000
Chronic-kidney disease					2.340 (1.230–3.410)	0.032
Congestive heart failure					1.333 (0.366–4.851)	0.662
Cerebrovascular disease						
Coronary-heart					2.894 (0.858–9.763)	0.087
Hypertension					9.397 (3.539–24.954)	< 0.000
Diabetes						

Abbreviations: OR, odds ratio; CI, confidence internal; NLR, neutrophil–lymphocyte ratio; CRP, c-reactive protein; pct, procalcitonin; BUN, blood urea nitrogen.

### Prediction of mortality by ROC curves

**Figure 3:**
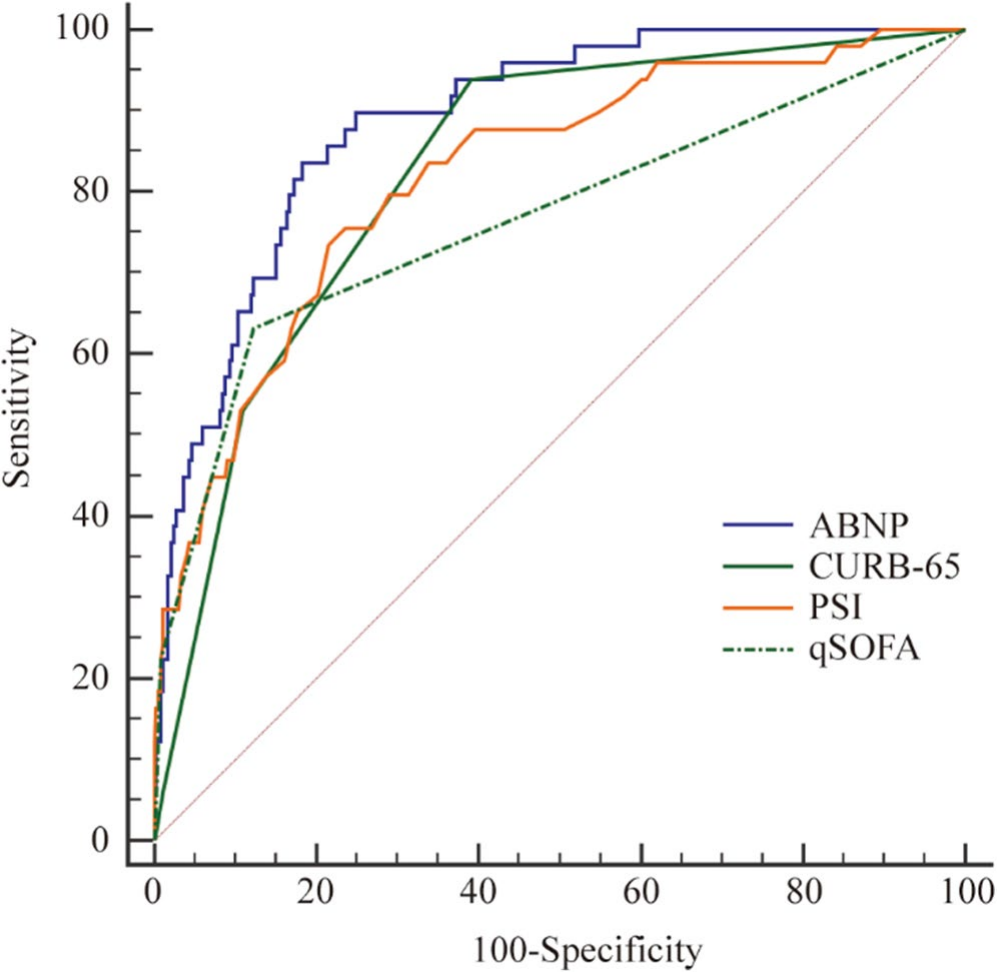
The Receiver Operating characteristic (ROC) curves of the four assessment scores for the mortality in group 1. The AUC of ABNP,CURB-65,PSI and qSOFA were 0.888, 0.827, 0.821 and 0.766, respectively.

**Figure 4:**
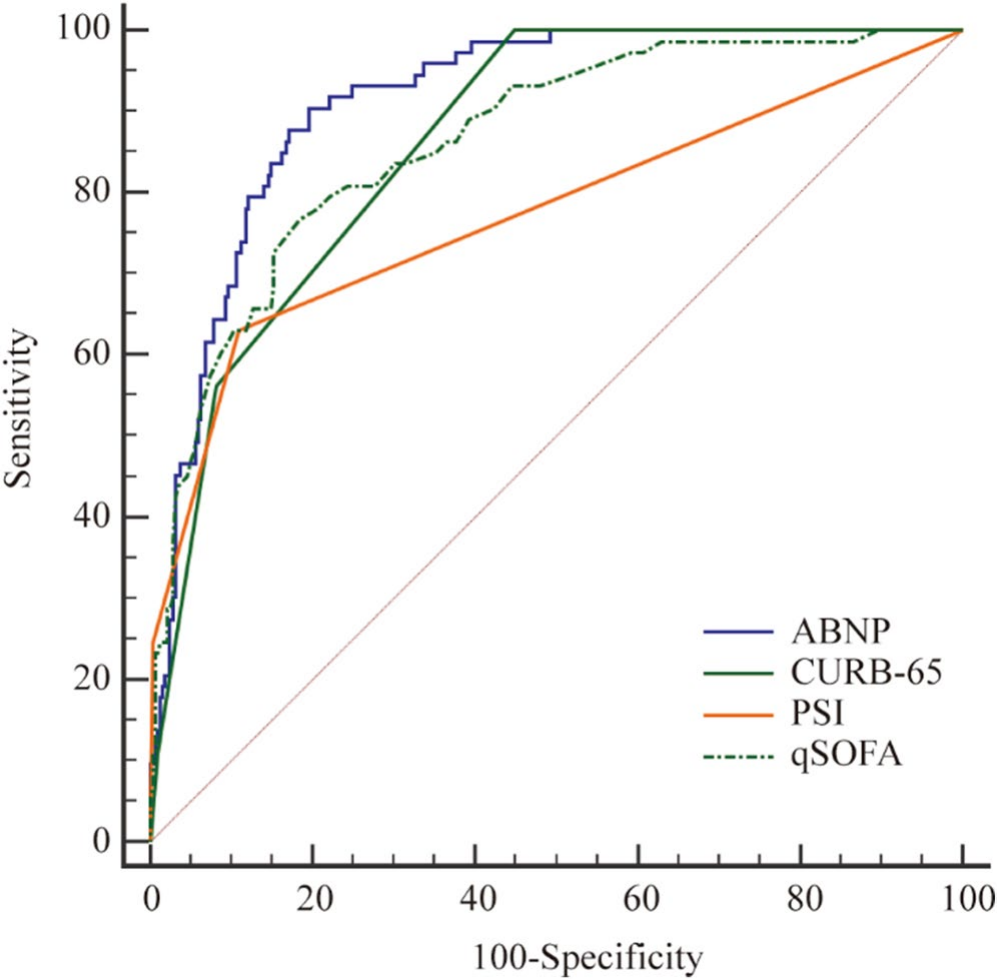
The Receiver Operating characteristic (ROC) curves of the four assessment scores for the mortality in group 2. The AUC of ABNP, CURB-65, PSI and qSOFA were 0.912, 0.863, 0.863 and 0.773, respectively.

**Figure 5:**
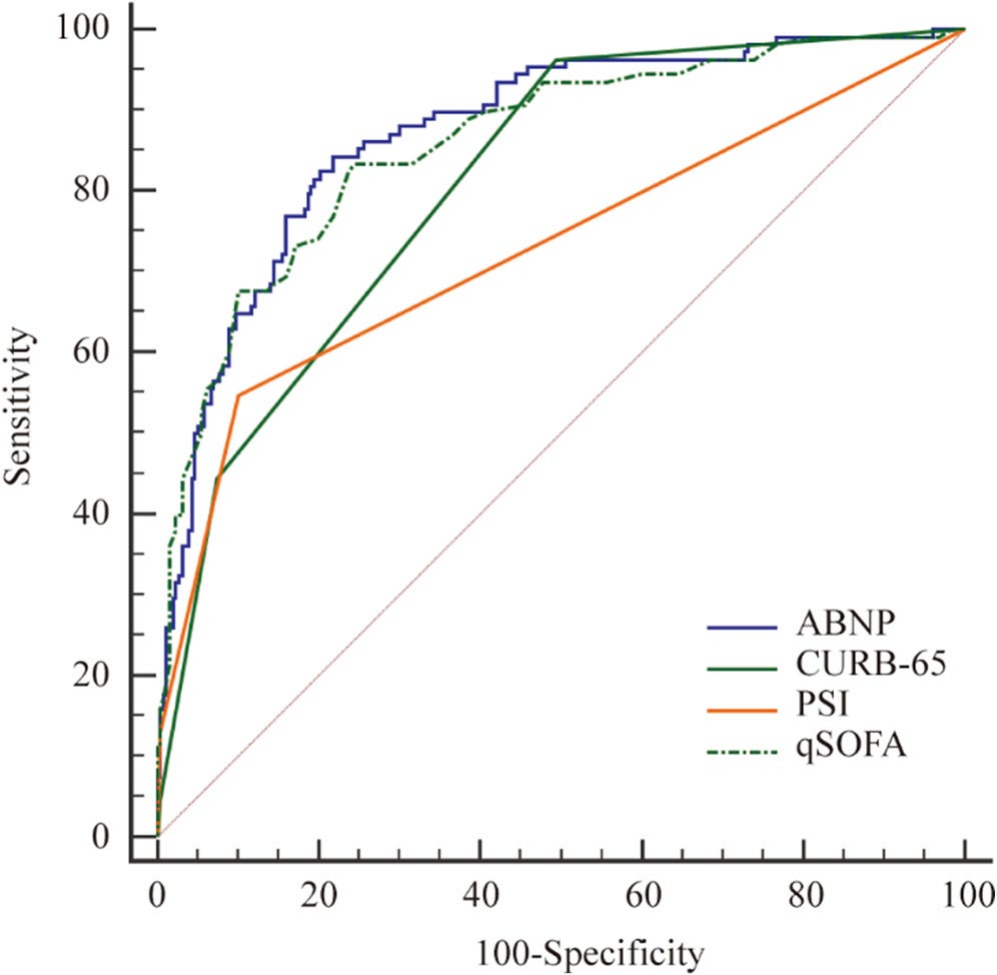
The Receiver Operating characteristic (ROC) curves of the four assessment scores for the mortality in group 3. The AUC of ABNP, CURB-65, PSI and qSOFA were 0.872, 0.809, 0.860 and 0.728, respectively.

Based on above finding, we believe it is reasonable to build a model by these 4 variables (as ABNP model) to predict the in-hospital mortality across all three groups. Further analysis showed that the AUC values of the ABNP model were 0.888 (95% CI 0.854–0.917, *p* < 0.000), 0.912 (95% CI 0.880–0.938, *p* < 0.000) and 0.872 (95% CI 0.833–0.905, *p* < 0.000) in group 1, 2 and 3, respectively (Table 3). As a comparison, we also calculated the AUCs of CURB-65, PSI and qSOFA in all the groups (Table 3) and compared them with the value from ABNP model in predicting in-hospital mortality (Table 4, Figs. 3, 4, 5). Interestingly, the results showed that ABNP model showed superior predictive efficiency when compared to CURB-65 (AUC = 0.827, *p* = 0.049)/ (AUC = 0.863, *p* = 0.008), PSI (AUC = 0.821, *p* = 0.045)/ (AUC = 0.863, *p* = 0.040) and qSOFA (AUC = 0.766, *p* = 0.004)/ (AUC = 0.773, *p* < 0.000) in both group 1 and 2, respectively. Moreover, in group 3, even though no significant difference was observed between ABNP model and PSI (AUC = 0.860, *p* = 0.060), our new established model (ABNP model) still showed better performance than CURB-65 (AUC = 0.809, *p* = 0.009) and qSOFA (AUC = 0.728, *p* < 0.00). Taken together, our results supported that our model (ABNP model) owns improved predictive capacity than clinically available models (including CURB-65, PSI and qSOFA).

**Table 3:** The AUC of ABNP and other assessment models in all groups.

Group	Assessment Scores	AUC	95%CI	*p* value
1	ABNP	0.888	0.854–0.917	< 0.000
	CURB-65	0.827	0.787–0.862	< 0.000
	PSI	0.821	0.781–0.857	< 0.000
	qSOFA	0.766	0.722–0.806	< 0.000
2	ABNP	0.912	0.880–0.938	< 0.000
	CURB-65	0.863	0.825–0.895	< 0.000
	PSI	0.863	0.826–0.896	< 0.000
	qSOFA	0.773	0.728–0.814	< 0.000
3	ABNP	0.872	0.833–0.905	< 0.000
	CURB-65	0.809	0.765–0.848	< 0.000
	PSI	0.860	0.819–0.893	< 0.000
	qSOFA	0.728	0.679–0.773	< 0.000

*Abbreviations*: AUC, area under the curve; CI, Confidence Interval; ABNP, Albumin + BUN + NLR + Pulse model; CURB-65, confusion, urea, respiratory rate, blood pressure, and ag ≥ 65 years; PSI, Pneumonia Severity Index; qSOFA, quick Sequential Organ Function Assessment.

**Table 4:** Comparison of ROC for mortality in three groups.

Group	Comparisons	Difference between areas	95% CI	z statistic	*p* value
1	ABNP vs. CURB-65	0.061	0.000–0.123	1.968	0.049
	ABNP vs.qSOFA	0.122	0.039–0.205	2.905	0.004
	ABNP vs.PSI	0.067	0.001–0.133	1.991	0.045
2	ABNP vs. CURB-65	0.049	0.012–0.085	2.642	0.008
	ABNP vs.qSOFA	0.139	0.075–0.203	4.254	< 0.000
	ABNP vs.PSI	0.048	0.002–0.095	2.050	0.040
3	ABNP vs. CURB-65	0.062	0.015–0.110	2.607	0.009
	ABNP vs.qSOFA	0.144	0.086–0.202	4.905	< 0.000
	ABNP vs.PSI	0.013	0.036–0.062	0.516	0.060

*Abbreviations*: ROC, Receiver Operating characteristic; CI, Confidence Interval; vs, versus; ABNP, Albumin + BUN + NLR + Pulse model; CURB-65, confusion, urea, respiratory rate, blood pressure, and ag ≥ 65 years; PSI, Pneumonia Severity Index; qSOFA, quick Sequential Organ Function Assessment.

## Discussion

Currently, there are few studies reporting the prognostic factors and assessment scores in different age-based subpopulations with CAP. This study, to our best knowledge, is the first study to understand adjusted parameters for prognosis across different aged groups in elderly CAP patients. Here, we divided elderly CAP patients into three groups classified by age and established a new model (ABNP model) based on parameters derived from prognostic values shared by all the age-dependent subgroups.

Consistent with previous studies, the mortality rates were 11.81%,18.53% and 29.59% in three groups, respectively, which showed the more pronounced mortality rate with increased age^19,20^. The highest mortality in CAP aged over 85 years group can be explained by the death-associated comorbidities. As shown in Table 1, we observed that comorbidities showed significantly difference between survivors and non-survivors in this group, including electrolyte disturbance, cancer, chronic kidney disease, congestive heart failure, coronary heart disease and hypertension. Moreover, several factors, like cancer, chronic kidney disease and hypertension, were still independent prognostic factors after the multiple logistic analysis. However, no comorbidities were significantly association with mortality in younger cohorts. This observation is accordant with previous studies of ours and others. For instance, we previously revealed that comorbidities were independent risk factors influencing in-hospital mortality in patients over 80 years old with CAP^21^. As well, Ghia et al. found comorbid conditions like chronic obstructive pulmonary disease, hypertension were common risk factors for CAP in the Indian population^22^. On this basis, we propose that more attention should be paid to the care of comorbidities in elderly patients with CAP, especially aged over 85.

Another interesting finding is that there are four variables (albumin, BUN, NLR and pulse) independently influencing prognosis in all three age-based subgroups. These variables have been studied and used in clinic. BUN and Pulse have been applied to some assessment scores, such as CURB-65 and PSI, and demonstrated to predict the prognosis of patients with CAP^23–26^. Additionally, although NLR and Albumin do not belong to common assessment score systems of CAP severity such as CURB-65 and PSI, we also found out NLR and Albumin can improve the predictive ability for mortality of elderly CAP, even in age-based elderly subgroups.

NLR, short for the ratio of absolute neutrophil count to absolute lymphocyte count, has also been identified to predict adverse outcome of patients with CAP^27–30^. Specifically, Cataudella et al.^27^ found NLR predicted 30-day mortality and performed better than PSI and CURB-65 score systems. Thirty-day mortality was 30% in those with a NLR between 11.12 and 13.4%, but 50% in those with a NLR between 13.4 and 28.3. Moreover, Feng et al.^31^ also discovered NLR was the independent factor influencing in-hospital mortality in elderly patients with CAP and showed higher AUC value than CURB-65 (0.72 vs. 0.678, *p* < 0.05). Therefore, a growing number of studies emphasize the importance of NLR to improve the ability of predicting adverse outcome in CAP patients when combined with other factors. A nomogram model composed by NLR was established by Lv et al.^32^ to predict mortality in elderly patients with CAP, and the AUC of the model was 0.9, which was proved to be superior to CURB-65 and PSI. Collectively, NLR is a simple, easily measured, yet promising marker for predicting outcomes in patients with CAP. Its value, either alone or in conjunction with other biomarkers, need to be further investigated.

The fourth variable worth attention is the serum albumin. Typically, albumin is well-known for its important roles in immune regulation and antimicrobial^33,34^. Accumulating evidence show that albumin is related to the prognosis of CAP patients. In one study, Sakakibara et al.^35^ established a new score model including albumin to predict severe adverse events (including death) in CAP patients, which exhibits a higher AUC value (0.85) compared with the other predictive models. Furthermore, another study by Shirata et al.^25^ developed another albumin-based system (using cutoff as 3.0 g/dL) to predict mortality in older patients with CAP, showing a higher AUC (0.809) than that of CURB-65. In addition, albumin decreased with aging for several potential reasons such as decline in cognition, poor oral health, and dysphagia^36,37^. Thus, it is necessary to increase the level of albumin in elderly patients with CAP, which may improve the prognosis. Several methods can be utilized, such as direct infusion of human albumin. Also, the nasogastric feeding was another preferable option to improve the albumin in elderly patients, especially in patients with decline in cognition after stroke, dysphagia and so on^38^. Finally, cumulative studies also support that the nasogastric feeding tube is efficient to deliver nutrients and/or fluids to the gastrointestinal tract effectively and play a central role in the management of elderly who were malnourished or hypoalbuminemia^39–42^.

Notably, the new established model (ABNP model) shows superiority over clinically used tools (CURB-65, PSI and qSOFA) regarding the prediction of mortality. Notably, the AUC of ABNP model was still higher than PSI score even though there is no significant difference between them in the subgroup aged over 85 years. This could possibly be explained by the contribution of comorbidities. Multiple logistic analysis showed that comorbidities (cancer, chronic-kidney disease and hypertension) were also independent variables influencing mortality in aged over 85 years patients, besides ABNP-associated factors (albumin, BUN, NLR and pulse). Taken as a whole, we arrived a conclusion that ABNP model was an improved scoring system for prognosis prediction in elder CAP patients.

There are some limitations. Firstly, this study is a single-center study, which leads to a limited number of samples and may even cause bias in sample collection. Thus, the results of this study should be verified in multi-center, large-sample studies in the future. Secondly, our study is a retrospective observational study. This may cause several issues, including the possible poor quality of available data due to undesigned study, the possible absence of important data on potential confounding factors and differential losses to follow up on study cohort. Therefore, prospective studies, if applicable, are essential to increase the reliability. Thirdly, other clinical factors are not taken into consideration, such as antibiotic therapy and pathogen infection, and some data are missing for individual patients, such as D-dimer and prealbumin. Fourthly, some data analyzed in this study might not show authentication. For instance, odds ratio values of hypertension and cancer in our analysis were inflated. This might be due to a limited number of samples with hypertension (or cancer) in survivor or non-survivor group. This situation may be related to the special conditions in a data set and this is known as “monotone likelihood”^43^. Thus, we will collect more patient data and apply reconstruction of the interval estimation based on profile penalized log likelihood (PPL) to solve this concern^44^. Finally, we did not take functional decline or frailty into account, which could influence the prognosis of patients with CAP in elderly patients^45–47^. Thus, more studies with large population need to be designed in the future.

## Conclusions

We established an early prediction model based on an age-group-specific study of elderly patients with CAP. The new model of the AUCs in predicting mortality in different age groups (66–75/ 76–85/ over 85 years) were higher than PSI, CURB-65 and qSOFA.

### Supplementary Information

Supplementary Figure 1.

Supplementary Information.

## Data Availability

All data generated or analysed during this study are included in this published article [and its supplementary information files].
