# Author Correction: Establishment of risk model for elderly CAP at different age stages: a single-center retrospective observational study

**DOI:** 10.1038/s41598-023-40951-7

**Published:** 2023-08-23

**Authors:** Chunxin Lv, Teng Pan, Wen Shi, Weixiong Peng, Yue Gao, Abdul Muhith, Yang Mu, Jiayi Xu, Jinhai Deng, Wei Wei

**Affiliations:** 1 Oncology Department, Shanghai Punan Hospital of Pudong New District, No 279, Linyi Road, Pudong, Shanghai, China; 2 Longgang District Maternity & Child Healthcare Hospital of Shenzhen City, Shenzhen, China; 3 Faculty of Life Sciences and Medicine, School of Cancer and Pharmaceutical Sciences, King’s College London, London, UK; 4 Department of Dermatology, Shanghai Punan Hospital of Pudong New District, No 279, Linyi Road, Shanghai, China; 5 Clinical Research Center (CRC), Medical Pathology Center (MPC), Cancer Early Detection and Treatment Center (CEDTC), Translational Medicine Research Center (TMRC), Chongqing University Three Gorges Hospital, Chongqing University, Wanzhou, Chongqing, China; 6 Department of Oncology, Royal Marsden Hospital, London, UK; 7 Geriatric Department, Minhang Hospital, Fudan University, No 170, Xinsong Road, Shanghai, China; 8 Richard Dimbleby Department of Cancer Research, Comprehensive Cancer Centre, Kings College London, London, SE1 1UL UK; 9 Hunan Zixing Artificial Intelligence Technology Group Co., Ltd., Hunan Province, Changsha City, China

Correction to: *Scientific Reports* https://doi.org/10.1038/s41598-023-39542-3, published online 01 August 2023

In the original version of this Article, Weixiong Peng, Yue Gao and Yang Mu were incorrectly affiliated with ‘Clinical Research Center (CRC), Medical Pathology Center (MPC), Cancer Early Detection and Treatment Center (CEDTC), Translational Medicine Research Center (TMRC), Chongqing University Three Gorges Hospital, Chongqing University, Wanzhou, Chongqing, China’. The correct affiliation is listed below.

‘Hunan Zixing Artificial Intelligence Technology Group Co., Ltd., Hunan Province, Changsha City, China’

The original Article has been corrected.

