# Supplementary figures and images for: Establishment of risk model for elderly CAP at different age stages: a single-center retrospective observational study
Source: Sci Rep. 2023 Aug 1;13:12432. doi: 10.1038/s41598-023-39542-3 (PMC10393957; doi:10.1038/s41598-023-39542-3)

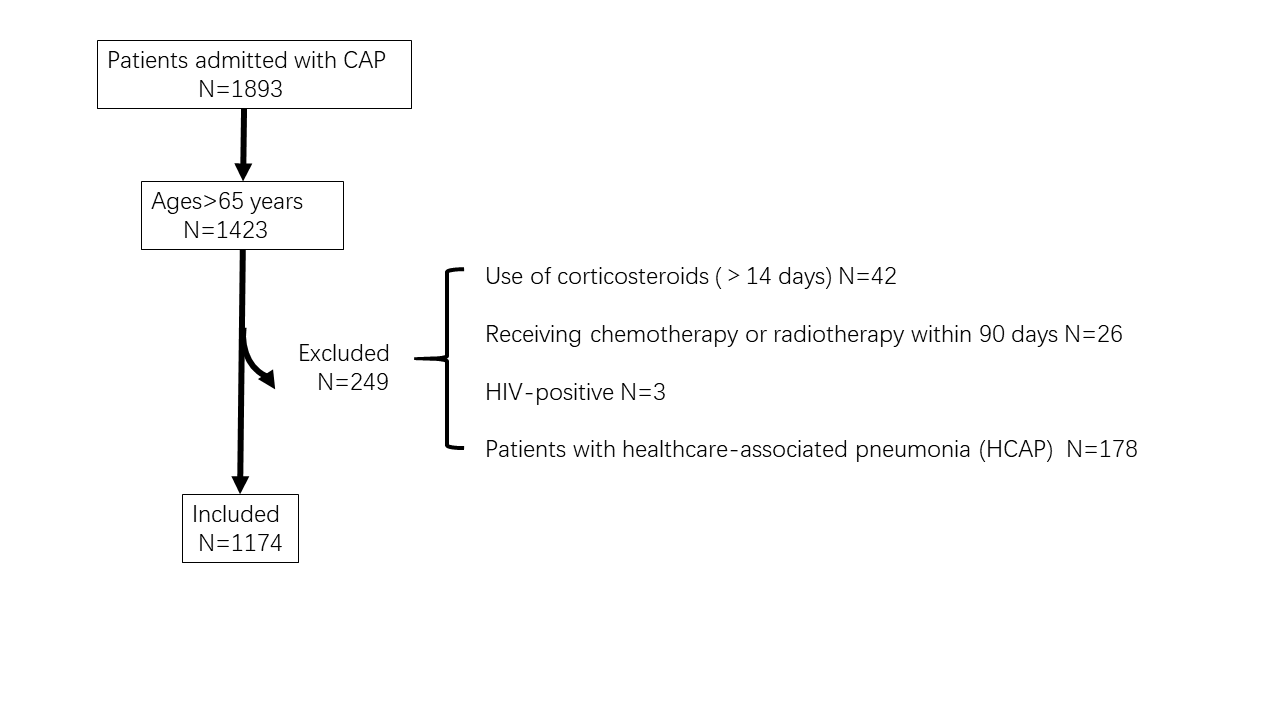


Supplementary Figure 1. Schematic diagram of patients selection.

Supplement: Supplementary file 1 — Supplementary Figure 1. [file 41598_2023_39542_MOESM1_ESM.docx]
